# Top 50 Most Cited Articles on Thoracic Ossification of Posterior Longitudinal Ligament

**DOI:** 10.3389/fsurg.2022.868706

**Published:** 2022-05-09

**Authors:** Xing Ding, Ming Yan, Jinze Wu, Chongqing Xu, Yinjie Yan, Zhixing Yu, Mengchen Yin, Jinhai Xu, Junming Ma, Wen Mo

**Affiliations:** Shanghai Longhua Hospital, Shanghai, China

**Keywords:** thoracic, OPLL, bibliometric analysis, most cited, top 50

## Abstract

**Study Design:**

Bibliometric analysis.

**Objective:**

Over the last several decades, the field of thoracic ossification of the posterior longitudinal ligament (T-OPLL) has evolved unprecedentedly, and the literature on T-OPLL has increased significantly. The purpose of this study is to identify and review the top 50 most cited publications related to T-OPLL.

**Methods:**

The most frequently cited 50 articles in this field until 30 October 2021 were identified by searching Web of Science. We ranked the articles based on the citation number. Through the bibliometric method, we evaluated the following information: article title, first author, year of publication, journal of publication, total number of citations, country, and study topic.

**Results:**

The number of citations of included studies ranged from 20 to 108, with a mean number of 45.4. The journal *Spine* published most articles (20), followed by *Spinal Cord* (5), and *European Spine* (5). All of these articles were contributed by 38 first authors, *Yamazaki* (4), *Fujimura* (3), and *Aizawa* (3) who published more than 2 articles. In the respect of productive countries, Japan (39) contributed most papers. Tomita contributed the most cited article in 1990 on *Spine*, which was the first-ever report of circumferential decompression for thoracic myelopathy due to T-OPLL.

**Conclusion:**

The top 50 influential articles on T-OPLL were identified and analyzed in this study. It will undoubtedly provide a comprehensive and detailed basis for the orthopedic and neurosurgery physicians to make a clinical decision and assimilate the research focus of spine surgery.

## Introduction

T-OPLL is the heterotopic ossification of the spinal ligament with unclear pathogenesis. T-OPLL is one of the common etiologies of thoracic myelopathy. According to *Matsumoto M*'s report, the incidence of T-OPLL was 0.8% approximately (1–3).

Surgical decompression is the only effective therapeutic method, and the surgical treatment of T-OPLL is the research focus in this field. Currently, a variety of surgical procedures have been developed, and the postoperative complication rate is 9.6–40.8% (4). Each surgical method has pros and cons, and the optimal surgical modalities are still a matter of contention.

The citation count is a useful surrogate measure of the scientific impact of a published work (5). Bibliometrics is the cross-disciplinary science of quantitative analysis of all knowledge carried by mathematical and statistical methods (6). Some bibliometric analysis on back pain and spinal disorders has been published, but, regretfully, the most cited papers regarding T-OPLL have not been analyzed in this method (7–9).

This study aimed to intuitively show the research focus and reported surgical procedures of T-OPLL, especially by analyzing the top 50 influential articles with the bibliometric method. We hope this study will provide a comprehensive and detailed basis for orthopedic and neurosurgery physicians.

## Methods

The data for this study were collected from the Web of Science (WOS) and its Core Collection on 30 October 2021. We used the following search command: posterior longitudinal ligament AND ossification AND thoracic. All English articles were published from January 1980 to October 2021 and confined to T-OPLL.

According to citation numbers, all sorted papers were ranked in descending order. Based on a title and an abstract, two independent reviewers confirmed their relevance to T-OPLL. The final results included original articles and reviews. For each paper, the following information was recorded and analyzed: title, author, country of origin, journal of publication, year of publication, and citations number.

## Results

The literature search yielded 202 articles. According to citation number, the top 50 highly cited articles were sorted in our study. The citation number ranged from 20 to 108, with an average citation number of 45.4, and the final 50 articles have received 2,270 total citations (Table 1). There were 18 articles that were cited more than 50 times.

**Table 1:** 50 Top-cited articles in the field of TOPLL.

**Rank**	**First author**	**Title**	**Journal**	**Total citations**	**Year**	**Average Citation**
1	Tomita, K	Circumspinal decompression for thoracic myelopathy due to combined ossification of the posterior longitudinal ligament and ligamentum flavum	Spine	108	1990	3.60
2	Yamazaki, M	Clinical results of surgery for thoracic myelopathy caused by ossification of the posterior longitudinal ligament: Operative indication of posterior decompression with instrumented fusion	Spine	92	2006	6.57
3	Aizawa, T	Results of surgical treatment for thoracic myelopathy: minimum 2-year follow-up study in 132 patients	Journal of Neurosurgery-Spine	89	2007	6.85
4	Matsumoto, M	Surgical results and related factors for ossification of posterior longitudinal ligament of the thoracic spine—A multi-institutional retrospective study	Spine	87	2008	7.25
5	Takahata, M	Clinical results and complications of circumferential spinal cord decompression through a single posterior approach for thoracic myelopathy caused by ossification of posterior longitudinal ligament	Spine	77	2008	6.42
6	Fujimura, Y	Long-term follow-up study of anterior decompression and fusion for thoracic myelopathy resulting from ossification of the posterior longitudinal ligament	Spine	71	1997	3.09
7	Matsuyama, Y	Surgical outcome of ossification of the posterior longitudinal ligament (OPLL) of the thoracic spine—Implication of the type of ossification and surgical options	Journal of Spinal Disorders & Techniques	69	2005	4.60
8	Tokuhashi, Y	Effectiveness of posterior decompression for patients with ossification of the posterior longitudinal ligament in the thoracic spine—Usefulness of the ossification-kyphosis angle on MRI	Spine	65	2006	4.64
9	Kawahara, N	Circumspinal decompression with dekyphosis stabilization for thoracic myelopathy due to ossification of the posterior longitudinal ligament	Spine	64	2008	5.33
10	Yamazaki, M	Posterior decompression with instrumented fusion for thoracic myelopathy caused by ossification of the posterior longitudinal ligament	European Spine Journal	58	2010	5.80
11	Sato, T	Thoracic myelopathy in the Japanese: Epidemiological and clinical observations on the cases in Miyagi prefecture	Tohoku Journal of Experimental Medicine	58	1998	2.64
12	Min, JH	Clinical results of ossification of the posterior longitudinal ligament (OPLL) of the thoracic spine treated by anterior decompression	Journal of Spinal Disorders & Techniques	57	2008	4.75
13	Tsuzuki, N	Staged spinal cord decompression through posterior approach for thoracic myelopathy caused by ossification of posterior longitudinal ligament	Spine	54	2001	2.84
14	Kawaguchi, Y	Association between polymorphism of the transforming growth factor-beta 1 gene with the radiologic characteristic and ossification of the posterior longitudinal ligament	Spine	53	2003	3.12
15	Matsumoto, M	Outcomes of fusion surgery for ossification of the posterior longitudinal ligament of the thoracic spine: a multicenter retrospective survey Clinical article	Journal of Neurosurgery-Spine	52	2011	5.78
16	Matsuyama, Y	Indirect posterior decompression with corrective fusion for ossification of the posterior longitudinal ligament of the thoracic spine: is it possible to predict the surgical results?	European Spine Journal	51	2009	4.64
17	Fujimori, T	Prevalence, Concomitance, and Distribution of Ossification of the Spinal Ligaments: Results of Whole Spine CT Scans in 1500 Japanese Patients	Spine	50	2016	12.50
18	Yamazaki, M	Transient paraparesis after laminectomy for thoracic ossification of the posterior longitudinal ligament and ossification of the ligamentum flavum	Spinal Cord	48	2006	3.43
19	Sharan, AD	Approaching the upper thoracic vertebrae without sternotomy or thoracotomy—A radiographic analysis with clinical application	Spine	48	2000	2.40
20	Yamazaki, M	Transient paraparesis after laminectomy for thoracic myelopathy due to ossification of the posterior longitudinal ligament—A case report	Spine	46	2005	3.07
21	Hanai, K	Anterior decompression for myelopathy resulting from thoracic ossification of the posterior longitudinal ligament	Spine	46	2002	2.56
22	Iwasawa, T	Pathophysiological role of endothelin in ectopic ossification of human spinal ligaments induced by mechanical stress	Calcified Tissue International	44	2006	3.14
23	Hou, XF	Clinical Features of Thoracic Spinal Stenosis-associated Myelopathy A Retrospective Analysis of 427 Cases	Clinical Spine Surgery	43	2016	10.75
24	Chang, UK	Surgical treatment for thoracic spinal stenosis	Spinal Cord	40	2001	2.11
25	Ono, M	Ossification of the thoracic posterior longitudinal ligament in a fixed population. Radiological and neurological manifestations	Radiology	40	1982	1.05
26	Stapleton, CJ	Ossification of the posterior longitudinal ligament: genetics and pathophysiology	Neurosurgical Focus	39	2011	4.33
27	Aizawa, T	Thoracic myelopathy in Japan: Epidemiological retrospective study in Miyagi Prefecture during 15 years	Tohoku Journal of Experimental Medicine	39	2006	2.79
28	Kurosa, Y	Selecting a surgical method for thoracic myelopathy caused by ossification of the posterior longitudinal ligament	Spine	39	1996	1.63
29	Kalb, S	Analysis of demographics, risk factors, clinical presentation, and surgical treatment modalities for the ossified posterior longitudinal ligament	Neurosurgical Focus	38	2011	4.22
30	Mori, K	Prevalence, Distribution, and Morphology of Thoracic Ossification of the Posterior Longitudinal Ligament in Japanese	Spine	34	2014	5.67
31	Epstein, NE	Ossification of the yellow ligament and spondylosis and or ossification of the posterior longitudinal ligament of the thoracic and lumbar spine	Journal of Spinal Disorders	34	1999	1.62
32	Fujimura, Y	Myelopathy secondary to ossification of the posterior longitudinal ligament of the thoracic spine treated by anterior decompression and bony fusion	Spinal Cord	34	1997	1.48
33	Li, M	Management of thoracic myelopathy caused by ossification of the posterior longitudinal ligament combined with ossification of the ligamentum flavum-a retrospective study	Spine Journal	33	2012	4.13
34	Zhang, HQ	Posterior decompression with kyphosis correction for thoracic myelopathy due to ossification of the ligamentum flavum and ossification of the posterior longitudinal ligament at the same level Clinical article	Journal of Neurosurgery-Spine	33	2010	3.30
35	Park, JY	Thoracic ligament ossification in patients with cervical ossification of the posterior longitudinal ligaments—Tandem ossification in the cervical and thoracic spine	Spine	32	2008	2.67
36	Kojima, T	Surgical treatment of ossification of the posterior longitudinal ligament in the thoracic spine	Neurosurgery	30	1994	1.15
37	Ikeda, Y	Association between serum leptin and bone metabolic markers, and the development of heterotopic ossification of the spinal ligament in female patients with ossification of the posterior longitudinal ligament	European Spine Journal	29	2011	3.22
38	Ohtani, K	Anterior surgical decompression for thoracic myelopathy as a result of ossification of the posterior longitudinal ligament	Clinical Orthopedics and Related Research	28	1982	0.74
39	Kawaguchi, Y	Ossification of the Posterior Longitudinal Ligament in Not Only the Cervical Spine, but Also Other Spinal Regions Analysis Using Multidetector Computed Tomography of the Whole Spine	Spine	27	2013	3.86
40	Takemoto, M	Additive-manufactured patient-specific titanium templates for thoracic pedicle screw placement: novel design with reduced contact area	European Spine Journal	26	2016	6.50
41	Tomita, K	Total decompression of the spinal cord for combined ossification of posterior longitudinal ligament and yellow ligament in the thoracic spine	Archives of Orthopedic and Trauma Surgery	26	1990	0.87
42	Hirai, T	Prevalence and Distribution of Ossified Lesions in the Whole Spine of Patients with Cervical Ossification of the Posterior Longitudinal Ligament A Multicenter Study (JOSL CT study)	Plos One	25	2016	6.25
43	Ido, K	Anterior decompression and fusion for ossification of posterior longitudinal ligament in the thoracic spine	Journal of Spinal Disorders	25	1995	1.00
44	Guo, QF	Simultaneous ossification of the posterior longitudinal ligament and ossification of the ligamentum flavum causing upper thoracic myelopathy in DISH: case report and literature review	European Spine Journal	22	2011	2.44
45	Aizawa, T	Sagittal alignment changes after thoracic laminectomy in adults	Journal of Neurosurgery-Spine	22	2008	1.83
46	Ido, K	Surgical treatment for ossification of the posterior longitudinal ligament and the yellow ligament in the thoracic and cervico-thoracic spine	Spinal Cord	21	1998	0.95
47	Fujimura, Y	Anterior decompression and fusion for ossification of the posterior longitudinal ligament of the upper thoracic spine causing myelopathy: Using the manubrium splitting approach	Spinal Cord	21	1996	0.88
48	Onishi, E	Outcomes of Surgical Treatment for Thoracic Myelopathy A Single-institutional Study of 73 Patients	Spine	20	2016	5.00
49	Nishida, N	Biomechanical study of the spinal cord in thoracic ossification of the posterior longitudinal ligament	Journal of Spinal Cord Medicine	20	2011	2.22
50	Cho, JY	Management of Cerebrospinal Fluid Leakage After Anterior Decompression for Ossification of Posterior Longitudinal Ligament in the Thoracic Spine The Utilization of a Volume-controlled Pseudomeningocele	Journal of Spinal Disorders & Techniques	18	2012	2.25

The top 50 highly cited articles were published by 16 different journals, while the journal with the largest share of publications was the journal *Spine* (20). *Spinal Cord* (5) and *European Spine* (5) had the second most publications. Eight journals contributed at least 2 papers, which had 42 total publications (Table 2).

**Table 2:** Journal distribution of the 50 top cited articles on TOPLL.

**Journal**	**Publications**
Spine	20
European Spine Journal	5
Spinal Cord	5
Journal of Neurosurgery-Spine	4
Journal of Spinal Disorders & Techniques	2
Neurosurgical Focus	2
Tohoku Journal of Experimental Medicine	2
Journal of Spinal Disorders	2
Calcified Tissue International	1
Clinical Spine Surgery	1
Radiology	1
Neurosurgery	1
Clinical Orthopedics and Related Research	1
Archives of Orthopedic and Trauma Surgery	1
Plos One	1
Journal of Spinal Cord Medicine	1

Overall, the 50 studies were contributed by 38 first authors, of whom 8 authors published more than one paper (Table 3). Yamazaki was the most productive author with four publications, followed by Fujimura and Aizawa, who published three articles. The highest number of total citations was still Yamazaki from Japan with an average of 61 citations per article.

**Table 3:** Authors with multiple publications.

**Author**	**Publications**
Yamazaki, M	4
Fujimura, Y	3
Aizawa, T	3
TOMITA, K	2
Matsuyama, Y	2
Matsumoto, M	2
Kawaguchi, Y	2
Ido, K	2

On the final list, there were only four countries (Table 4): Japan, the USA, China, and South Korea. The majority of identified papers were contributed by Japan (39). According to the list, Japan was the most influential country in the field of T-OPLL over the past four decades. Four papers originated from China, and USA, and Korea that contributed to three publications.

**Table 4:** Countries of the 50 top cited articles on TOPLL.

**Country**	**Publications**
Japan	39
USA	4
China	4
Korea	3

The total number of institutions responsible for the top 50 cited articles was 36, and 5 of them contributed at least 2 articles (Table 5). The most affiliated institutions were in Japan, which was similar to the analysis of the productivity of countries. *Chiba University Graduate School of Medicine* had the highest number of most cited articles (5), followed by *Keio University* with four papers.

**Table 5:** Institutions with multiple publications of the 50 top cited articles on TOPLL.

**Institution**	**Publications**
Chiba University Graduate School of Medicine	5
Keio University	4
Kanazawa University	3
Tohoku University School of Medicine	3
Nagoya University School of Medicine	2

**Figure 1:**
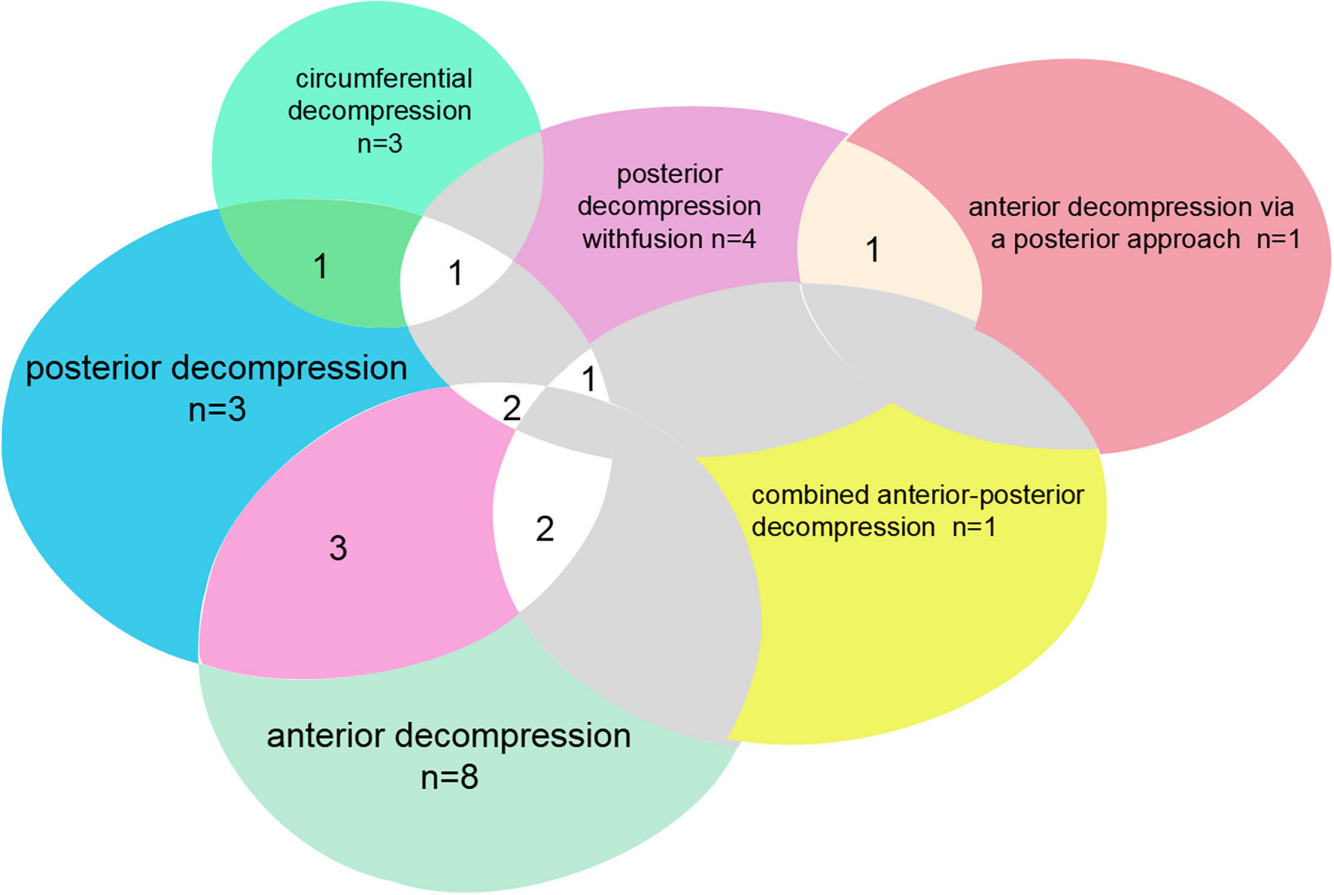
Distribution and intersection points of the top 50 cited articles on surgical type on TOPLL.

Regarding the study topics, the majority of these 50 articles were related to surgical treatment (31), 21 articles focused on the study of a specific surgical procedure type, and the rest of the articles related to surgery could be categorized as analyses of the surgical treatment's prognostic factors and selection of different surgical methods. There are 11 studies that included more than one surgical treatment, and intersection clusters were formed in these procedures (Figure 1). Besides surgical treatments, other topics mainly included clinical features and prevalence (8), pathophysiology (5), and biomechanical (2). When we analyzed these articles according to the types of study design, 37 articles could be classified as clinical research (including randomized trials, cohort studies, retrospective studies, etc.), 5 were epidemiologic surveys, 3 were case reports, 3 were pathological mechanism studies, 2 were radiology studies, and 1 was a 3D model study. Among them, clinical research accounted for the largest proportion. This was because the most common study topic was surgical treatment—the only effective clinical method for T-OPLL.

The two earliest articles were published in 1982. One was written by Ono et al. and published in *Radiology*, which was the first epidemiologic survey of the prevalence of T-OPLL by a decade in a fixed population sample (10). The other one was written by *Ohtani* and published on *Clinical Orthopedics and Related Research*, a case report of anterior surgical decompression for T-OPLL (11). The most recent papers were five articles published in 2016 (12–16). Notably, one paper published in 2016 written by *Takemoto* introduced novel-designed image-based navigational patient-specific templates for pedicle screw placement, which reduced the contact area on the bone without sacrificing the stability of the template (14). The concept of this design shed light on the field of spine surgery.

## Discussion

About 70% of OPLL occurs in the cervical spine and 15% in the thoracic spine (17). Although T-OPLL is a subject of relatively few published studies for the moderate incidence, it usually has an insidious onset, a long disease course, and a very high rate of disability. Through bibliometric analysis, this study aims to evaluate the most-cited articles in the literature, focusing on T-OPLL and reaching a conclusion with several implications for future research and practice.

Ossification of posterior longitudinal ligament occurs most frequently in East Asian populations, which may explain why three of the four productive countries on the final list are East Asian countries (18). According to the *Mori*'s epidemiologic survey, CT-based prevalence of T-OPLL in the Japanese population was 1.9% (19). Because of the regional limitations of this disease, five identified articles were epidemiologic surveys conducted in Japan. The USA was the only non-East Asian country on the list with four publications. Research from the USA involved many respects of the spinal field. With an extremely low incidence in this country, T-OPLL was also studied by USA scholars.

As further analysis was conducted of the identified articles, we found that the five most cited articles' study topics were all related to surgical treatment. Owing to the narrow canal, the rigidity of the thoracic spine, tenuous blood supply, and the inability of the spinal cord to withstand much compression, the myelopathy symptoms of T-OPLL are often severe, and surgical treatment is inevitable (20). With the unique anatomy and pathophysiology characteristics of the thoracic spine, T-OPLL surgery is one of the most challenging surgeries in orthopedics. However, there is still a lack of unified standards for surgical approach selections (4, 21, 22). Spine surgeons have been exploring ideal operative treatments for T-OPLL over the last several decades.

The most cited article was a case report published by Tomita et al. with 108 citations in 1990, which was the first report of circumferential compression (CM) for T-OPLL (23). The author found that the ratio of thoracic myelopathy due to combined OPLL and ossification of the ligamentum flavum (OLF) was low when laminectomy was used alone. OPLL combined with OLF led to circumferential compression of the spinal cord in advanced stages of the disease. CM consisted of two steps: posterior and lateral decompression of the spinal cord by removal of the OLF and anterior removal of the OPLL for anterior decompression. About 10 patients received CM, and the ratio of recovery was between 56 and 100%. Although the study was published in earlier years, this landmark study pioneered the use of the CM technique for TOPLL, and CM was intensively studied and modified in several later studies (21, 24, 25). The advent of this radical but effective procedure was significant progress in the field.

Yamzaki et al. conducted a retrospective study in 2006, which was ranked second on the final list (26). Notably, this study was the most cited paper on comparative analysis of the different surgical modalities. This research was compared with the clinical outcomes of posterior decompression and fusion, posterior decompression, and OPLL extirpation for thoracic myelopathy caused by OPLL. This study indicated that posterior decompression with instrumented fusion obtained a considerable degree of neurologic recovery with an extremely low rate of postoperative complications.

The third most cited article was by Aizawa et al. (26), which was published in 2007 (27), a follow-up study that investigated the results of surgical treatments in 132 patients with thoracic myelopathy and 31 patients with T-OPLL among them. About 24 patients with laminectomy had the worst postoperative JOA score, as the spinal cord could hardly shift backward enough when laminectomy alone was used. This study also found that patients with milder myelopathy and a shorter duration of myelopathy before surgery had better neurological improvement after decompressive surgery.

Postoperative complication has been a constant challenge in surgical procedures, and we found four of the five most cited articles compared the complications according to the type of surgery. A recent systematic review of complications in surgery for T-OPLL has indicated that the indirect decompression approach (posterior decompression and posterior decompression with dekyphosis) was relatively safer than the direct decompression approach (anterior decompression, posterior circumferential decompression, and combined anterior and posterior decompression) (28). This study retrieved 15 studies, including a total of 595 patients. The indirect decompression approach had a lower overall incidence of complications (16.1 vs. 45.4%; *p* < 0.001) and better neurologic function recovery postoperatively (63.4 vs. 44.4%; *p* = 0.073) than the direct decompression approach, which supports the result of Yamzaki et al's. retrospective study (26) in 2006—the second most cited article. In the existing literature, various surgical procedures have been reported to treat this difficult pathology. Ultimately, in devising a surgical management strategy, it will be dependent on the preference and comfort level of the surgeon. While some lesions may be tackled from anterior or circumferential approaches, it appears that most authors favor a posterior approach to the treatment of T-OPLL (29). With the modern pedicle screw instrumentation system, a posterior-only, indirect approach may be sufficient to decompress the spinal cord from the OPLL.

When OPLL was combined with OLF, severe thoracic myelopathy occurred. In our study, we found circumferential decompression, laminectomy, and posterior decompression with fusion are the main surgical methods in the identified articles. The results of most studies showed that circumferential decompression performs well at neurologic recovery with a high rate of complications and posterior decompression with fusion obtains a considerable degree of neurologic recovery with a relatively low rate of complications. Yamazaki et al. reported a case (30) with T-OPLL combined with OLF, in which postoperative paralysis occurred after laminectomy and was reversed after an additional posterior instrumented fusion. Laminectomy is indirect and the simplest method for T-OPLL decompression, but postoperative paraparesis is the main drawback of this technique. Through the review of these studies, we found posterior decompression with fusion was a safe surgical solution to the condition of T-OPLL combined with OLF. As revealed in Tomita et al's. first report of circumferential decompression (23), it was an effective but radical surgical procedure.

As, for clinical features, multiple identified studies pointed out that thoracic myelopathy caused by T-OPLL can present acutely after minor trauma, and prognostic factors for poor outcomes include longer preoperative duration of symptoms, worse preoperative symptoms, OPLL and/or OLF, a large volume of intraoperative bleeding, and diabetes mellitus. When we made the comparison in each bleeding volume of the same type of surgical procedure in these identified studies, we could not find clear differences in the bleeding volume of recent surgeries and that of surgeries in earlier times. For example, the average bleeding volumes of circumferential decompression (CM) in Tomita et al. first report (23) and Kawahara's case report published in 2008 were 3,190 and 2,900 ml, respectively. We argue that this cannot reflect the absence of advancement in surgical techniques. According to the analysis of identified articles' study topics, we can find spine surgeons have been modifying surgical methods and refining surgery concepts to make more accurate treatment decisions, which have largely ameliorated the surgical prognosis of T-OPLL.

Except for surgery treatment and population incidence, more studies focused on the genetic level of OPLL and documented many genes or gene loci of interest involved in mediating the molecular and genetic pathobiology of OPLL, including COL6A1, COL11A2, TGFβ-1, and IL17RC (31, 32). As neurological surgeons achieved a deeper understanding of the genetic and molecular pathogenesis of OPLL, therapies targeted at preventing the initial formation and progression of T-OPLL may be pursued further.

Citation analysis was a commonly used bibliometric tool to analyze scientific literature with several possible limitations (33). First, only publications indexed by WOS were analyzed; only part of known journals was indexed in WOS. As a result, some T-OPLL publications were missed or not included in this analysis. Second, as articles were identified according to the number of citations, some new significant publications in the field have no had enough opportunity to be cited by other authors. Their novel techniques and ideas may be ignored. Therefore, it tended to be a kind of retrospective study of historical articles. Thirdly, only published journal papers were included in this analysis. Other papers like clinical guidelines, meeting notes, and textbooks were excluded, which may have a greater impact in this field. Fourth, some authors had opinions that the citation number could not reflect the quality of research necessarily, with the drawbacks like self-citation, language bias toward English, and statements of the competitor (34, 35). Finally, some studies with a considerably minor proportion of T-OPLL cases were also included in our analysis. We identified these papers from all of the retrieved articles and finally decided to enroll these articles into our study after thoughtful consideration, because we purpose to conduct a comprehensive and detailed bibliometric analysis of T-OPLL, and these papers are indispensable.

## Conclusion

This study highlighted the top 50 most cited articles in the field of T-OPLL, including the article title, first author, year of publication, journal of publication, total number of citations, country, and study topic. In terms of study topics, surgical treatments accounted for the largest proportion, which has been developing over the last several decades. The majority of the study design was clinical research, followed by epidemiologic surveys. In addition, several novel and instructive studies may be missed in our study for the inherent limitation of bibliometric analysis. In summary, our study provided a comprehensive and detailed basis for orthopedic and neurosurgery physicians, and we expected the appearance of more high-quality research, which could promote the development of the field of T-OPLL.

## Data Availability Statement

The original contributions presented in the study are included in the article/supplementary material, further inquiries can be directed to the corresponding author/s.

## Author Contributions

XD and MYa are contributing to the design, conduct of the study, and drafting the manuscript. All authors read and approved the final manuscript.

## Funding

This work was supported by Shanghai Municipal Health Commission (2021LPTD-008), Shanghai Science and Technology Commission (21S21900500), and the Fifth Batch of Longhua Hospital Affiliated to Shanghai University of Traditional Chinese Medicine (KC2022006).

## Conflict of Interest

The authors declare that the research was conducted in the absence of any commercial or financial relationships that could be construed as a potential conflict of interest.

## Publisher's Note

All claims expressed in this article are solely those of the authors and do not necessarily represent those of their affiliated organizations, or those of the publisher, the editors and the reviewers. Any product that may be evaluated in this article, or claim that may be made by its manufacturer, is not guaranteed or endorsed by the publisher.

## Abbreviations

T-OPLL: thoracic ossification of posterior longitudinal ligament.
